# Influence of angiotensin-converting enzyme inhibition on reversibility of alterations in arterial wall and cognitive performance associated with early hypertension: A follow-up study

**DOI:** 10.1097/MD.0000000000016966

**Published:** 2019-08-23

**Authors:** Enikő Csikai, Mónika Andrejkovics, Bernadett Balajthy-Hidegh, Gergely Hofgárt, László Kardos, Ágnes Diószegi, Róbert Rostás, Katalin Réka Czuriga-Kovács, Éva Csongrádi, László Csiba

**Affiliations:** a Institute of Behavioural Sciences, Faculty of Public Health; b Department of Neurology, Faculty of Medicine, University of Debrecen; c Kenézy Gyula University Hospital; d Department of Medicine, Faculty of Medicine, University of Debrecen; e MTA-DE Cerebrovascular and Neurodegenerative Research Group, Debrecen, Hungary.

**Keywords:** arterial stiffness, carotid intima-media thickness, cognition, flow-mediated vasodilatation, hypertension

## Abstract

The importance of optimal blood pressure control for preventing or reducing the impairment of vascular and cognitive functions is well known. However, the reversibility of early alterations in vascular and cognitive functions through antihypertensive agents is under-investigated. In this study, we evaluated the influence of 3 months of angiotensin-converting enzyme (ACE) inhibition treatment on the morphological and functional arterial wall and cognitive performance changes in 30 newly diagnosed primary hypertensive patients.

Common carotid intima-media thickness (IMT) and brachial artery flow-mediated dilatation (FMD) were detected by ultrasonography. Arterial stiffness indicated by augmentation index (AIx) and pulse wave velocity (PWV) was assessed by arteriography. Cognitive functions were assessed by neuropsychological examination.

The executive function overall score was significantly higher at 3-month follow-up than at baseline (median, 0.233 (IQR, 0.447) vs –0.038 (0.936); *P* = .001). Three-month ACE inhibition did not produce significant improvement in IMT, FMD, AIx and PWV values. Significant negative associations were revealed between IMT and complex attention (*r* = –0.598, *P* = .0008), executive function (*r* = –0.617, *P* = .0005), and immediate memory (*r* = –0.420, *P* = .026) overall scores at follow-up. AIx had significant negative correlations with complex attention (*r* = –0.568, *P* = .001), executive function (*r* = –0.374, *P* = .046), and immediate memory (*r* = –0.507, *P* = .005). PWV correlated significantly and negatively with complex attention (*r* = –0.490, *P* = .007).

Timely and effective antihypertensive therapy with ACE inhibitors has significant beneficial effects on cognitive performance in as few as 3 months. Early ACE inhibition may have an important role in the reversal of initial impairments of cognitive function associated with hypertension-induced vascular alterations.

## 1 Introduction

Worldwide, cardio- and cerebrovascular diseases are identified as leading causes of death, and are attributable to the common underlying mechanism of atherosclerosis.^[1]^ Hypertension is one of the most important independent risk factors of atherosclerosis.^[2]^ The vascular system is a predilection site of hypertension-related damage. Endothelial dysfunction is the initial pathophysiological step in vascular damage.^[3]^ A previous prospective study showed that forearm endothelial dysfunction determined by FMD is highly predictive for cardiovascular morbidity and future cardiovascular events in initially untreated and uncomplicated subjects with essential hypertension.^[4]^ A dysfunctional endothelium may lose its ability to protect the vascular system through a loss of its anti-atherosclerotic and antithrombotic potential, thus playing a key pathophysiological role in the development and progression of the atherosclerotic process. Carotid IMT, a morphological characteristic of the arterial wall, is considered to be a reliable marker of preclinical atherosclerosis.^[5]^ Carotid IMT correlates with the severity of hypertension and its growth is a good indicator of atherosclerosis progression.^[6]^ Moreover, carotid IMT is a predictive factor for future vascular events.^[7]^ Arterial stiffness, a functional arterial wall feature described via AIx and PWV, is an early sign of atherosclerosis.^[8]^ Increased arterial stiffness is an independent predictor of cardiovascular events and all-cause mortality.^[9]^

The impact of hypertension on vascular remodeling is a well-known phenomenon, but consequential cognitive impairment has been debated in the scientific literature. The American Heart Association published a scientific statement in 2016 which stated that there were insufficient data to make evidence-based recommendations.^[10]^ However, the debate is far from over, as a recently published systematic review specified the MoCA as the recommended tool for differentiating vascular dementia from vascular mild cognitive impairment.^[11]^ Both publications emphasized the need for further research, with the following emphasis from the 2016 AHA publication: “Antihypertensive drugs are generally safe and widely available, but there is still much to be learned about how to best use them over the life course in the presence of comorbidities and whether specific classes of drugs may confer cognitive benefits beyond BP lowering.”^[10]^

Substantial evidence supports a link between hypertension and cognition.^[12,13]^ This relationship might be mediated by vascular system impairment. Hypertension disrupts the structure of cerebral blood vessels, promotes atherosclerosis, and impairs vital cerebrovascular regulatory mechanisms. Hypertension has been associated with executive dysfunction, reduced mental processing speed, and, less frequently, memory deficits.^[14]^ Although cognitive impairment is well documented in people with hypertension, little is known about the reversibility of cognitive changes in this population.^[13]^

The renin-angiotensin-aldosterone system (RAAS) has a central role in the pathophysiology of hypertension as well as cardio- and cerebrovascular diseases evolving from atherosclerosis.^[15,16]^ It is well known that by blocking the RAAS, ACE inhibitors possess, in addition to blood pressure-lowering effects, a vascular protective and anti-ischemic action through their anti-atherosclerotic, antithrombotic, and anti-inflammatory effects.^[17,18]^ A previous study has produced evidence that ACE inhibition is an emerging potential modality of cognitive protection by minimizing irreversible brain and heart damage.^[19]^ However, the influence of ACE inhibition on the reversibility of initial atherosclerotic vascular alterations and cognitive performance impairments induced by early-stage hypertension is still under investigation. Therefore, in this 3-month follow-up study, we aimed to evaluate the influence of ACE inhibitors on early morphological and functional changes of the arterial wall and cognitive performance impairments in newly diagnosed primary hypertensive patients. We also investigated the associations between initial atherosclerotic arterial wall alterations and cognitive function parameters.

## 2 Methods

### 2.1 Patients

**Figure 1:**
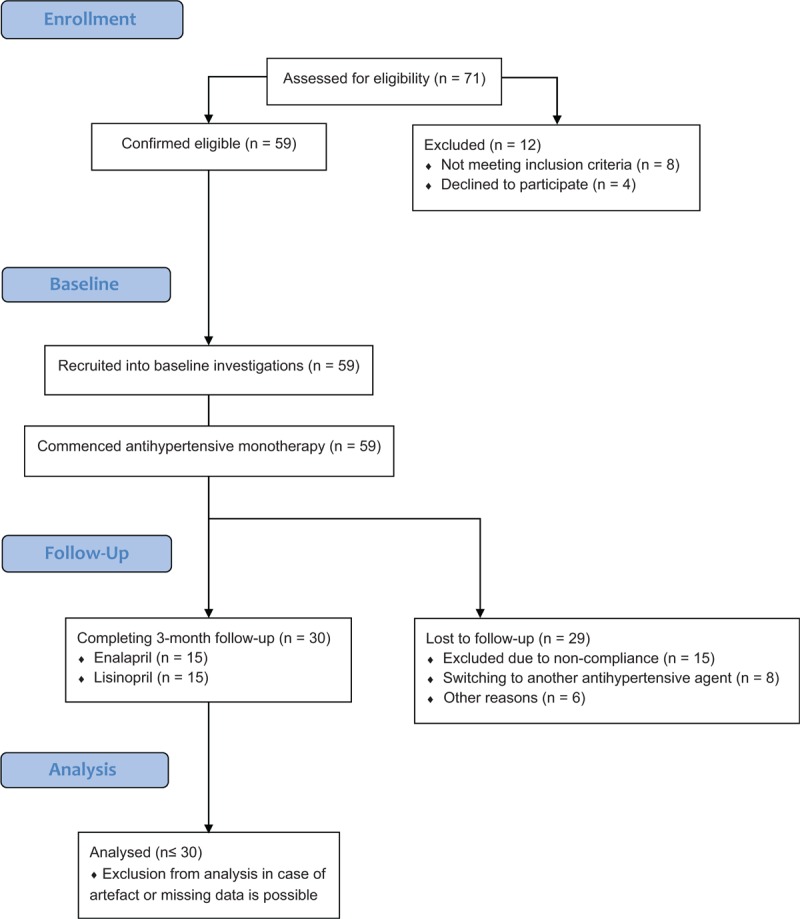
Flow diagram of the study.

From January 2014 to January 2017, 59 patients with recently diagnosed primary hypertension were recruited into our baseline investigations (Fig. 1). No participants were on antihypertensive treatment at baseline measurements. After baseline investigations, antihypertensive monotherapy was commenced with an angiotensin-converting enzyme inhibitor (enalapril or lisinopril). Twenty-nine patients had been excluded from the study secondary to non-compliance or switching from ACE inhibitor therapy to another antihypertensive agent due to side effects or inefficacy. Hence, the final data analysis involved 30 patients (age: 43.60 ± 11.34 years; male/female ratio: 2.0, body mass index: 27.81 ± 4.04 kg/m^2^) who completed the follow-up visit at three months. No subjects were pregnant or suffered from malignancy, impaired liver or renal function, alcohol or drug dependence, infectious diseases, or symptomatic cerebro- or cardiovascular diseases as observed through medical history, general physical and neurological examination, routine laboratory tests, and cerebral computed tomography scan. There were 5 smokers among the study participants. Thirty percent of patients had higher education. The study protocol was approved by the Ethics Committee of the University of Debrecen and the study was carried out in accordance with the Declaration of Helsinki. All participants gave written informed consent.

### 2.2 Laboratory assays

Venous blood samples were collected after overnight fasting. Serum ions, basic kidney functions, glucose levels, lipid profile, and complete blood count were assayed by routine automated laboratory methods. High-sensitivity C-reactive protein was assessed by turbidimetric assay on an Integra 800 analyzer (Roche Diagnostics, Mannheim, Germany). Hemoglobin A1C was measured by high-performance liquid chromatography (BioRad, Hercules, CA). Fibrinogen concentration was determined by the Clauss method.

### 2.3 Ambulatory blood pressure monitoring

Twenty-four-hour ambulatory blood pressure monitoring (ABPM) was performed using the ABPM-04 device (Meditech Ltd., Budapest, Hungary). Blood pressure was recorded every 15 minutes during the daytime (6 am through 10 pm) and every 30 minutes during nighttime (10 pm through 6 am). Based on ABPM data, mean systolic and diastolic blood pressures and systolic and diastolic hyperbaric indices for daytime and nighttime were determined.

### 2.4 Common carotid intima-media thickness measurement

Philips HD 11 XE ultrasound equipment with a 7.5-MHz linear transducer was used to measure common carotid intima-media thickness (IMT). Online measurements of IMT were performed in the far artery wall of the common carotid arteries, 10 mm proximal to the carotid bulb. IMT was determined as the distance between the lumen-intima interface and the upper layer of the adventitia. All measurements were performed on frozen, enlarged images at end-diastole, with the transducer in the mediolateral direction. Ten measurements of IMT were performed on both sides. The mean of the 20 IMT values in each patient was calculated.

### 2.5 Brachial artery flow-mediated dilatation measurement

Brachial artery flow-mediated dilatation (FMD) assessment was performed using the HP Sonos 5500 ultrasound with 10-MHz linear assay transducer. A B-mode longitudinal section was obtained from the brachial artery above the antecubital fossa. A forearm cuff was inflated to supra-systolic pressures for 5 minutes to induce arterial occlusion. Upon cuff release, the induced reactive hyperemia promotes an increase in shear stress-mediated NO release and subsequent vasodilation. FMD is expressed as the percent increase in arterial diameter following cuff release with the arterial diameter at baseline as reference.

### 2.6 Assessment of arterial stiffness

Measurements were carried out using a TensioClinic arteriograph (TensioMed Ltd., Hungary). Arterial stiffness was assessed by determining the augmentation index (AIx) and pulse wave velocity (PWV). The method is based on the phenomenon of myocardial contractions generating pulse waves in the aorta. The pulse wave travels to the arm (first wave), where the cuff is located, then back to the aorta. The first wave is reflected at the bifurcation of the aortic wall; therefore, a second, reflected wave appears as a late systolic peak. The cuff detects both pulse waves. AIx is calculated from the amplitudes of the first and second wave and represents the pressure difference between the late systolic peak and the early systolic peak divided by the pulse pressure. PWV is the ratio of the jugular fossa–symphysis distance and the reflection time at 35-mmHg suprasystolic pressure on the brachial artery.

### 2.7 Neuropsychological assessment

All patients underwent a comprehensive 1.5-hour long neuropsychological examination carried out and evaluated by trained psychologists. The test battery was composed specifically for the determination of the main neurocognitive functions as listed by the Diagnostic and Statistical Manual of Mental Disorders, Fifth Edition (*DSM*-*5*): reaction time, attention, executive function, learning, memory, and perceptual-motor skills. The battery included the most sensitive tests to reveal minor cognitive function deviations, which are not necessarily evident during everyday activity. The categorization shown in Table 3 was used to assign each test of the battery to 1 of 3 neuropsychological domains of importance in hypertension-related cognitive impairment.

**Table 3:**
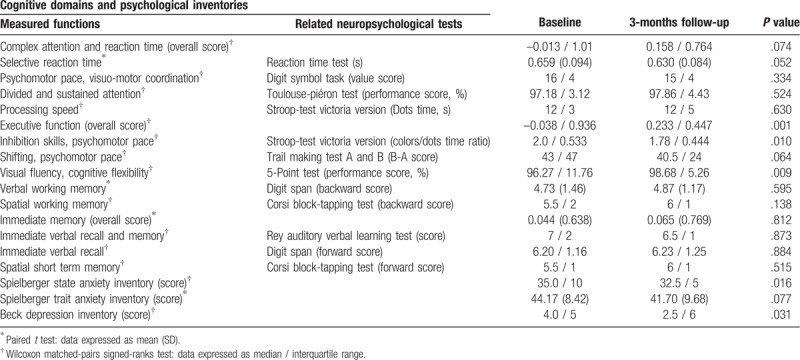
Neuropsychological tests and questionnaires (n = 30).

Because anxiety and depression assessment is a basic requirement in neurocognitive examination, the subjects filled out the State-Trait Anxiety Inventory and the Beck Depression Inventory; this enabled us to also examine if higher levels of these variables have a negative influence on performance.

### 2.8 Statistical analysis

Statistical analysis was performed using Stata version 15 (StataCorp LLC, College Station, TX). *P* < .05 was regarded as statistically significant. Baseline and follow-up levels of variables were described using standard statistics and compared using paired *t* tests if normality assumptions were satisfied, or Wilcoxon matched-pairs signed-ranks tests otherwise. Location and variability parameters mentioned in the body text are given as mean ± standard deviation or median (interquartile range).

Overall scores for each neuropsychological domain were derived by direction-correcting each variable in the domain (i.e., multiplying by negative 1 if necessary, so that greater values represent better performance), standardizing them, and taking their average.

### 2.9 Correlation estimates were based on Pearson's correlation coefficient

Associations between clinical data and neuropsychological outcomes were evaluated using linear regression. The outcome variable was additive change from baseline to follow-up in the neuropsychological parameter. Explanatory variables included baseline value and additive change in the clinical variable (key explanatory variables), an interaction term between them, age, sex, and baseline of the neuropsychological parameter. Model-predicted outcomes were plotted as a function of the key explanatory variables to assess which combination of the latter was predictive of significant changes in the outcome. Statistical proof for the relevance of the clinical parameter was interpreted through the joint *P* value associated with the key variable pair (general indication of relevance) as well as through the *P* value associated with the interaction term (indicating an interplay between baseline and change, i.e., a positive change from a low baseline has a different effect than one from an already high baseline, and likewise for negative changes).

## 3 Results

### 3.1 Laboratory and ABPM data

The laboratory characteristics of study participants at baseline and after 3 months of ACE inhibitor therapy are summarized in Table 1. There were no significant differences at 3-month follow-up compared to baseline in traditional vascular risk parameters, renal function, or complete blood count. ABPM data are demonstrated in Table 2. ABPM revealed a significant decrease in systolic and diastolic blood pressures and in systolic hyperbaric index for both active (daytime) and passive (nighttime) evaluations. However, no significant differences were detected between baseline and follow up in daytime or nighttime diastolic hyperbaric index.

**Table 1:**
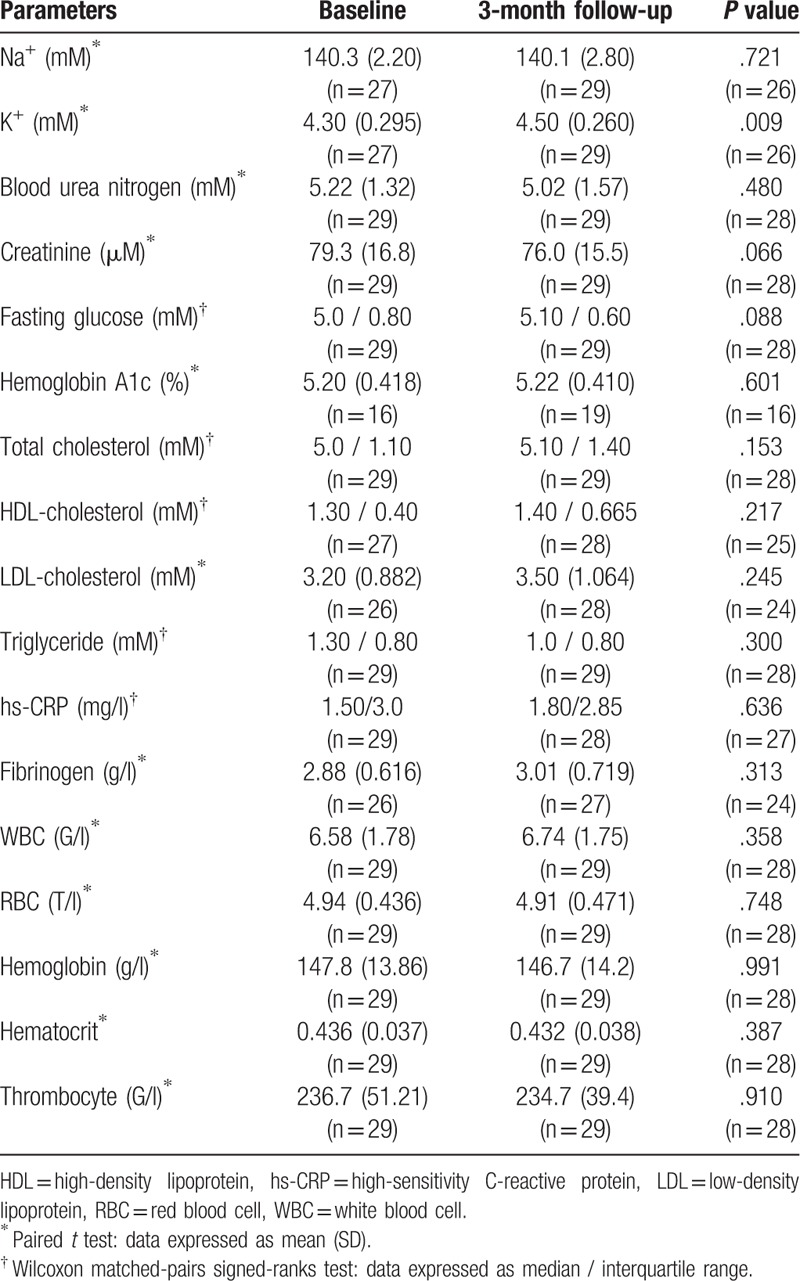
Laboratory parameters of study participants.

**Table 2:**
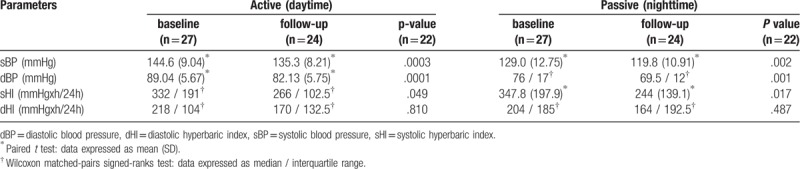
Ambulatory blood pressure monitoring data.

### 3.2 Arterial wall morphology and functional characteristics

No significant decrease in the IMT value was observed after 3 months of ACEI therapy (0.55 ± 0.10 mm at baseline vs 0.54 ± 0.08 mm at follow-up, *P* = .125). There was no significant increase in the FMD value after 3-month ACE inhibition (7.52 ± 2.21% at baseline vs 8.12 ± 2.60% at follow-up, *P* = .393). Arterial stiffness parameters also did not differ significantly between baseline and 3-month follow-up (AIx: –17.47 ± 34.44% vs –23.09 ± 34.32%, *P* = .078; PWV: 9.3 ± 2.40 m/s vs 9.0 ± 2.04 m/s, *P* = .141).

### 3.3 Neuropsychological tests and questionnaires

The neuropsychological test results are given in Table 3. After three months of ACEI therapy, significant improvement was revealed in executive function (–0.038 (0.936) vs 0.233 (0.447); *P* = .001); study patients had a significantly better performance in visual fluency (5-Point Test, 96.27 (11.76) vs 98.68 (5.26); *P* = .009) and inhibition skills (Stroop Test Victoria Version, 2.0 (0.533) vs 1.78 (0.444); *P* = .010). Complex attention and immediate memory overall scores did not show a significant improvement from baseline to follow up. The study participants reached significantly lower scores on the tests evaluating anxiety (Spielberger State Anxiety Inventory, *P* = .016) and depression (Beck Depression Inventory, *P* = .031) at follow-up compared to baseline.

We analyzed the relationships between the morphological and functional characteristics of the arterial wall and the parameters of cognitive functions by Pearson correlation analysis. Significant negative correlations were identified between IMT and complex attention (*r* = –0.482, *P* = .008 at baseline; *r* = –0.598, *P* = .0008 at follow-up), executive function (*r* = –0.420, *P* = .006 at baseline; *r* = –0.617, *P* = .0005 at follow-up) and immediate memory (*r* = –0.420, *P* = .026 at follow-up) overall scores. AIx had significant and negative correlations with complex attention (*r* = –0.410, *P* = .027 at baseline; *r* = –0.568, *P* = .001 at follow-up), executive function (*r* = –0.441, *P* = .017 at baseline; *r* = –0.374, *P* = .046 at follow-up), and immediate memory (*r* = –0.507, *P* = .005 at follow-up) overall scores. Significant and negative univariate correlation of PWV with complex attention overall score at follow up (*r* = –0.490, *P* = .007) was found, but a positive association of PWV with state anxiety score at follow-up (*r* = 0.397, *P* = .035) was detected.

FMD was correlated significantly and negatively with Spielberger Trait Anxiety score at baseline (*r* = –0.606, *P* = .001).

In evaluating the associations between ABPM data and neurocognitive performance overall test scores, systolic blood pressure correlated significantly and negatively with immediate memory overall score at baseline (*r* = –0.409, *P* = .034). Furthermore, the systolic hyperbaric index had significant and negative relationships with immediate memory overall score (*r* = –0.436, *P* = .023) and executive function overall score (*r* = –0.475, *P* = .012) at baseline.

Analyzing the associations of anxiety and depression levels with overall test scores, state anxiety showed significant and negative correlations with immediate memory at baseline (*r* = –0.413, *P* = .023) and executive function at follow up (*r* = –0.398, *P* = .030).

**Figure 2:**
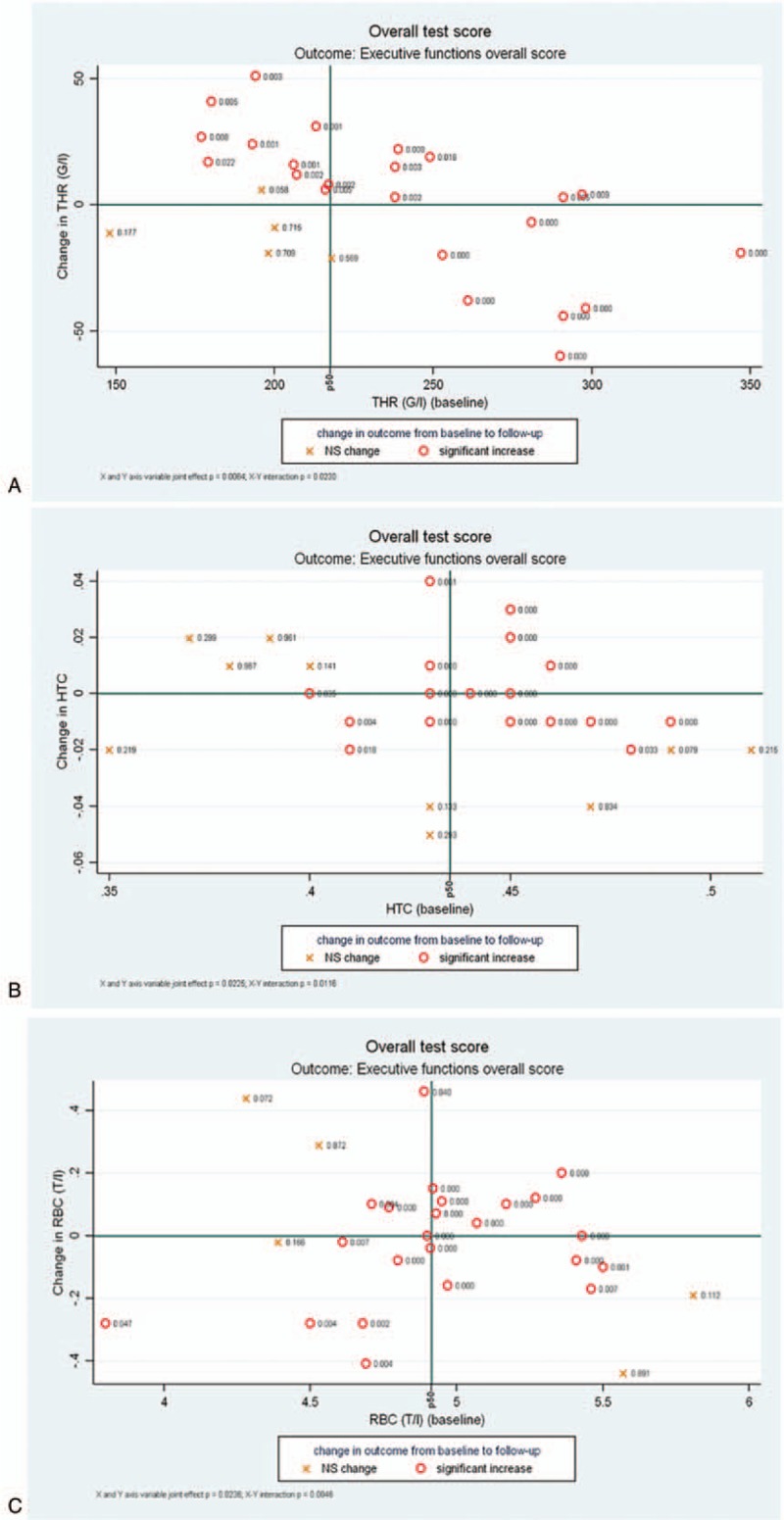
Executive function overall score changes from baseline to follow-up as a function of changes in, and baseline levels of, thrombocyte count (A), hematocrit (B), and red blood cell count (C). Marker labels indicate *P* value of model-predicted change at that location. HTC = hematocrit, NS = not significant, RBC = red blood cell, THR = thrombocyte.

The improvement in executive function overall score were associated with baseline thrombocyte count and change in thrombocyte count (joint effect *P* = .006 and interaction *P* = .023) presented in Figure 2(A), with baseline hematocrit values and change in hematocrit values (joint effect *P* = .023, interaction *P* = .012) presented in Figure 2(B), and also with baseline red blood cell count and change in red blood cell count (joint effect *P* = .024, interaction *P* = .005) presented in Figure 2(C).

## 4 Discussion

Since many of the more advanced vascular lesions in hypertension are not completely reversible, the importance of prevention, early treatment, and more effective blood pressure control is clearly demonstrated.^[20,21]^ However, the crucial issue concerning the reversibility of hypertension-induced early vascular alterations and consequential changed cognitive functions remained unanswered in critical aspects. By reducing the harmful effects of angiotensin II, RAAS blockade with an ACE inhibitor might provide a rational approach to reverse early hypertension-induced vascular damage and cognitive alterations.^[3,22]^ In this follow-up study, we investigated the effects of three months of ACE inhibition on hypertension-induced initial vascular and cognitive changes.

Abnormal endothelial function, arterial stiffness, and carotid media thickening have been implicated in the pathophysiology of essential hypertension.^[4,6,8]^ These morphological and functional characteristics of vascular damage in hypertensive patients can be detected with non-invasive techniques such as brachial FMD and carotid IMT with ultrasound, and arterial stiffness parameters (AIx, PWV) with arteriograph.

Miyamoto et al found that ACE inhibitor and angiotensin II receptor blocker (ARB) antihypertensive treatment can improve FMD-assessed endothelial dysfunction better than other drug types.^[23]^ In the present study, 3-month ACE inhibitor therapy has not resulted in significant changes in brachial artery FMD in hypertensive patients. Blood pressure-related media thickening is a manifestation of structural arterial wall remodeling in hypertensive patients.^[24]^ Puato et al showed that well-controlled blood pressure levels can prevent pro-atherogenic carotid artery remodeling.^[25]^

On the other hand, in the study by Ohta et al, carotid IMT increased progressively in spite of well-controlled home blood pressure values.^[26]^ After vascular injury, a significant amount of early neointimal dendritic cells showed angiotensin-II receptor expression. Tuleta et al found that even short-term ACE inhibitor therapy may diminish remodeling processes in injured vessels by a significant reduction of neointimal growth.^[27]^ Our research group reported previously that 1-year antihypertensive therapy results in significant improvement in vascular wall structure demonstrated by the reduction in carotid IMT.^[28]^ In our present study, the reductive effect of 3-month ACE inhibition on carotid IMT did not reach a significant level.

All classes of antihypertensive drugs might potentially decrease arterial stiffness passively via the reduction of the distending pressure.^[29,30]^ Results from clinical and experimental studies have suggested that activation of the RAAS may contribute to the development of arterial stiffness, and RAAS blockade in patients with hypertension has more pronounced effects on arterial stiffness than other antihypertensive drugs.^[31,32]^ Mallareddy et al performed a meta-analysis of clinical trials investigating the effects of ACE inhibitors on arterial stiffness measured by PWV or AIx, and concluded that ACEI have modest beneficial effects in reducing arterial stiffness, and this effect is at least partly independent of blood pressure changes.^[33]^ In this study, ACE inhibitor treatment for 3 months did not result in a significant decrease in arterial stiffness parameters.

Chronic arterial hypertension is a major contributor to cognitive impairment.^[34]^ The evidence to date points strongly to a deleterious influence of midlife hypertension on cognitive function in midlife and late-life. Executive function and processing speed seem to be the cognitive domains most affected, but memory can also be impaired.^[13]^ Whereas cognitive impairment is well documented in people with hypertension, several key questions remain to be answered regarding the impact of antihypertensive treatment on cognitive change in this population.^[13]^ After a long follow-up, an obvious benefit of antihypertensive therapy was demonstrated on cognitive functions.^[35]^ However, to date, there are limited data on the effect of shorter-term antihypertensive treatment on cognitive functions. In a study by Hanon et al, 6 months of ARB treatment, and in an investigation by Hajjar et al, 1 year of ARB therapy resulted in significant cognitive function improvement.^[36,37]^ Muldoon et al reported that short-term treatment with various antihypertensive drugs slightly improved working memory.^[38]^ A previous investigation by our study group showed that after 1 year of appropriate and effective antihypertensive therapy, normalized blood pressure was accompanied by an improved cognitive performance.^[28]^ Although some studies provided hints that certain classes of antihypertensive drugs may be more effective than others at improving cognition or reducing cognitive decline,^[39–41]^ most of these studies were underpowered or without equivalent cognitive endpoints.^[13]^ The current study demonstrated that the applied short-term (3-month) antihypertensive treatment with ACE inhibitors resulted in a significant improvement in executive function.

The association between hypertension and anxiety is well-recognized.^[42]^ However, the effect of antihypertensive treatment on anxiety is less clear and still remains controversial. Muldoon et al published that 6 weeks of antihypertensive treatment did not affect anxiety in middle-aged hypertensive patients.^[38]^ In contrast, patients with early hypertension reached significantly lower anxiety levels in the previous investigation of our research team after 1 year of antihypertensive therapy, and in this study after three months of ACE inhibitor treatment. Previous data are contradictory regarding the relationship between hypertension and depression. Scalco et al reported earlier that hypertension was associated with depression.^[43]^ Conversely, a meta-analysis of prospective cohort studies found no evidence that hypertension would be a risk factor for depression.^[44]^ Recently, the RAAS was proposed as being implicated in depression.^[45]^ There is increasing evidence (largely from animal models) for antidepressant and antianxiety effects of drugs targeting the RAAS.^[46–49]^ Our data provide evidence that depression level after 3-month-long ACE inhibition decreases significantly in patients with newly diagnosed hypertension.

Carotid intima-media thickness and arterial stiffness may serve as risk markers for vascular cognitive impairments.^[34]^ Previous studies observed significant inverse relationships of carotid IMT and arterial stiffness with cognitive function.^[34,50,51]^ In evaluating the associations of structural and functional artery characteristics with cognitive parameters in hypertensive patients, we found that carotid IMT and AIx correlated significantly and negatively with executive function, complex attention, and immediate memory. Furthermore, PWV had a significant negative relationship with complex attention. Moreover, this study has detected a significant positive correlation between PWV and anxiety, while FMD showed a significant negative association with anxiety. In addition, we established significant negative associations of anxiety with executive function and immediate memory. Thus, our data provide further evidence that markers of arterial wall alterations are closely associated with cognitive function in hypertension.

As less evidence is available on the relationship between blood cell count and cognitive function,^[52–54]^ the current study analyzed the associations between these parameters, trying to provide relevant information about the impact of blood cell count on cognitive performance. In the present investigation, for the first time in the literature, we have revealed significant positive associations of thrombocyte count, hematocrit, red blood cell count, and their changes after 3-month ACE inhibition with executive function in hypertensive patients. However, further aspects remain to be explored in this field.

A limitation of our study is the potential selection bias that might exist due to the fact that the Department of Neurology was recruiting untreated hypertensive patients for this study. In most cases, hypertension is diagnosed in primary care in Hungary; this study, however, involved patients referred to us by primary care on suspicion of hypertension, which was to be confirmed by our team through a diagnostic workup (ambulatory BP monitoring etc). Self-selection of patients included in the study is also possible since more than half of eligible patients were lost during the follow-up period. This results in a relatively small size of the study sample, which limits the statistical power of our investigation; however, the significant improvement in cognitive alteration caused by early hypertension-related vascular lesions underlines the positive impact of ACE inhibition on vascular deteriorations and consequential cognitive impairments. Furthermore, the measurements of morphological and functional characteristics of the arterial wall and the assessments of cognitive function parameters were performed after a short period of ACE inhibitor treatment. We, therefore, cannot provide information about what duration of ACE inhibition is required for significant improvement to arterial wall alterations.

## 5 Conclusion

In conclusion, timely and effective antihypertensive therapy with ACE inhibitors for as short a period as 3 months was observed to produce significant improvements in cognitive performance compromised by early hypertension-related vascular damage. Our study suggests that ACE inhibitors might serve as a potential agent for the reversal of cognitive alterations induced by initial vascular damage associated with early-stage hypertension. As to longer-term treatment, further prospective data are essential to determine the duration of ACE inhibition required for significant improvement in early hypertension-related vascular damage.

## Acknowledgments

The authors owe special thanks to our colleague Ildikó Borók József Ferencné for conducting medical diagnostic measurements and sonography.

## Author contributions

**Conceptualization:** Mónika Andrejkovics, László Csiba.

**Data curation:** Enikő Csikai, Bernadett Balajthy-Hidegh.

**Formal analysis:** Katalin Réka Czuriga-Kovács, László Csiba.

**Funding acquisition:** László Csiba.

**Investigation:** Enikő Csikai, Bernadett Balajthy-Hidegh, Gergely Hofgárt, Ágnes Diószegi, Róbert Rostás.

**Methodology:** Mónika Andrejkovics, László Kardos.

**Project administration:** Enikő Csikai, Bernadett Balajthy-Hidegh.

**Resources:** László Csiba.

**Supervision:** Mónika Andrejkovics, László Csiba.

**Validation:** László Kardos.

**Visualization:** László Kardos.

**Writing – original draft:** Enikő Csikai, László Kardos, Éva Csongrádi.

**Writing – review & editing:** Katalin Réka Czuriga-Kovács, László Csiba.
